# Development of nucleic acid lateral flow immunoassay for duplex detection of *Leishmania martiniquensis* and *Leishmania orientalis* in asymptomatic patients with HIV

**DOI:** 10.1371/journal.pone.0307601

**Published:** 2024-08-26

**Authors:** Namfon Nawattanapaibool, Toon Ruang-areerate, Phunlerd Piyaraj, Saovanee Leelayoova, Mathirut Mungthin, Suradej Siripattanapipong

**Affiliations:** 1 Department of Microbiology, Faculty of Science, Mahidol University, Bangkok, Thailand; 2 Department of Parasitology, Phramongkutklao College of Medicine, Bangkok, Thailand; Iran University of Medical Sciences, ISLAMIC REPUBLIC OF IRAN

## Abstract

Leishmaniasis, a neglected tropical disease caused by parasitic protozoa of the *Leishmania* genus, remains a global health concern with significant morbidity and mortality. In Thailand, the rising incidence of autochthonous leishmaniasis cases involving *Leishmania* (*Mundinia*) *martiniquensis* and novel *Leishmania* (*Mundinia*) *orientalis* underscores the critical need for accurate diagnosis and effective control strategies. This study presents a sensitive and specific nucleic acid lateral flow immunoassay (NALFIA) that integrates a duplex PCR assay with a lateral flow device (LFD) strip format. Targeting the internal transcribed spacer 1 (ITS1) region, known for its unique combination of conserved and variable sequences, this assay employs primers labeled with biotin, digoxigenin, and fluorescein isothiocyanate (FITC) markers, enabling precise species identification and differentiation of these two *Leishmania* species. Remarkably, the assay achieves a sensitivity that surpasses agarose gel electrophoresis, detecting as few as 10^−2^ parasite/μL for *L*. *martiniquensis* and 10^−4^ parasite/μL for *L*. *orientalis*. Notably, the assay exhibited reliable specificity, revealing no cross-amplification with other major viscerotropic *Leishmania* species or reference organisms. Evaluation using 62 clinical samples further confirms the effectiveness of the PCR-LFD assay, with a sensitivity of 100% for *L*. *martiniquensis* and 83.3% for *L*. *orientalis*, and an excellent agreement (*κ* value = 0.948) with nested PCR. This integrated assay represents a promising advancement in diagnostic tools, offering rapid and accurate results that can significantly contribute to effective disease management and control. Given the increasing relevance of these *Leishmania* species in current public health scenarios, this assay serves as a valuable tool for both diagnostic and research applications.

## Introduction

Leishmaniasis, caused by protozoan parasites of the *Leishmania* genus and transmitted through sandfly bites, remains a significant global health concern. With an estimated 12 to 15 million infections worldwide and 1.5 to 2 million new cases annually, it leads to approximately 70,000 deaths each year [1]. This disease exhibits diverse clinical manifestations, including skin lesions (cutaneous leishmaniasis—CL), mucous membrane involvement leading to tissue destruction (mucocutaneous leishmaniasis—MCL), and potentially life-threatening which affect visceral organs (visceral leishmaniasis- VL), depending on the *Leishmania* species infected [2, 3].

In Thailand, there has been a notable rise in indigenous cases involving two newly identified *Leishmania* species: *L*. (*Mundinia*) *martiniquensis*, a rare species initially discovered on Martinique Island [4], and *L*. (*Mundinia*) *orientalis*, a previously unknown species first reported in Thailand [5–7]. These species are responsible for causing both CL and VL in Thailand [8]. *Leishmania* typically circulates among various natural reservoir hosts and phlebotomine sandflies. However, recent field investigations and experimental findings have suggested that *Culicoides* biting midges (Diptera: Ceratopogonidae) might serve as competent vectors for transmitting *Leishmania* parasites of the subgenus *Mundinia*, potentially acting as primary vectors for indigenous leishmaniasis in Thailand. In addition, the co-circulation of *Crithidia* trypanosomatid with these two emerging *Mundinia* species in *Culicoides* biting midges suggests the possibility of this trypanosomatid co-transmission alongside *Leishmania* parasites. Understanding the prevalence and biology of *Crithidia* in these vectors would be crucial for a comprehensive assessment of leishmaniasis transmission dynamics [9–11].

Significantly, the number of leishmaniasis cases has been steadily increasing, particularly among immunocompetent and immunocompromised patients. Predominantly reported in southern and northern Thailand, approximately 40% of cases occurred in individuals with HIV/AIDS, a high-risk group for *Leishmania* infection [8, 12, 13]. Studies have shown a notable seroprevalence of *Leishmania* infection among HIV/AIDS patients, with many cases being asymptomatic [14]. A study at Trang Hospital found that 12% of patients tested positive for *L*. *orientalis* and 4.6% for *L*. *martiniquensis*, highlighting their prevalence among HIV patients [15]. The increasing indigenous cases among high-risk groups like patients with HIV/AIDS emphasize the need for effective disease prevention and control measures. Having a prompt and accurate diagnosis is crucial for emerging *Leishmania* infections.

The leishmaniasis diagnosis, especially VL, primarily relies on immunological and molecular approaches [16]. Immunological tests such as the rK39 rapid diagnostic test (rK39 RDT) and the Direct Agglutination Test (DAT) have been considered suitable options for diagnosing VL in humans [17]. However, the rK39 RDT has some restrictions, as it cannot detect antibodies against *L*. *martiniquensis* and *L*. *orientalis*. A recombinant antigen derived from *L*. *martiniquensis*-k39 has been developed to overcome this limitation, showing promising sensitivity. Nonetheless, cross-immunoreaction with sera positive for *L*. *donovani* and *L*. *infantum* suggested some degree of antigenic similarity among these species [18]. Moreover, antibody-based tests cannot differentiate between current and past infections, rendering them unsuitable for treatment prognosis assessment, particularly in immunocompromised individuals such as those with HIV/AIDS, where antibody production may be reduced or delayed. Additionally, most of molecular-confirmed cases are asymptomatic and often present with varying levels of antibody titers [14, 19].

Given these limitations, antigen-based tests or molecular methods such as PCR are often advisable, as these methods directly detect the parasite or its components, indicating the ongoing infection, which provides more reliable results. PCR is a precise and sensitive tool for diagnosing leishmaniasis by targeting multiple genomic regions for detection [20–22]. Of these, the internal transcribed spacer 1 (ITS1), spacer DNA located between the 18S and 5.8S rRNA genes within the small subunit ribosomal RNA (SSU-rRNA), has been one of the common genetic markers with highly conserved properties and variable sequences, enabling the identification of different *Leishmania* species [23–26]. In contrast to PCR, the loop-mediated isothermal amplification (LAMP) assay requires less procedure and non-instrument nucleic acid amplification (NINA), which is comparatively cheaper than PCR [13, 27, 28]. Furthermore, the LAMP could simultaneously interpret and semi-quantify the *Leishmania* infection levels and others in a one-step assay [29–31]. However, while PCR and LAMP targeting the ITS1-rRNA region have been developed to detect *L*. *martiniquensis* and *L*. *orientalis*, they could not differentiate between *Leishmania* species without sequencing [32], which highlights the limitations of currently available diagnostic tools in accurately distinguishing between these closely related species.

This study proposed to develop a cutting-edge innovative approach: a species-specific PCR targeting the ITS1 region, combined with the nucleic acid lateral flow immunoassay (NALFIA) using a user-friendly lateral flow device (LFD) strip format. The integration of NALFIA represents a significant advancement in diagnostic technology, combining the principles of lateral flow technology and immunoassays to detect nucleic acids with exceptional efficiency [33, 34]. This approach eliminates the need for labor-intensive gel electrophoresis and the costly equipment associated with real-time PCR machines traditionally used to detect amplified DNA targets. Moreover, it can simultaneously detect multiple target amplicons by labeling PCR amplicons and capturing those using immobilized antibodies on LFDs, thus providing species discrimination of *L*. *martiniquensis* and *L*. *orientalis*.

## Materials and methods

### Ethics statement

All experimental protocols and methods were approved by Mahidol University’s Central Institutional Review Board (Protocol No. MU-CIRB 2021/282.2705). This study used archived samples, and the data were fully anonymized before being accessed on August 10, 2022. The requirement for informed consent was waived, a decision approved by the IRB.

### Sample preparation and DNA extraction

For *Leishmania* parasites culture, the promastigote stage of *L*. *martiniquensis* (MON-229; MHOM/TH/2011/PG) and *L*. *orientalis* (*L*. *siamensis*, MON-324; MHOM/TH/2010/TR) were cultured using Schneider’s Drosophila medium (Sigma-Aldrich, USA) supplemented with 20% fetal bovine serum (FBS) (Gibco, UK), whereas *L*. *donovani* (MHOM/IN/1983/AG83), and *L*. *infantum* (MHOM/TN/80/IPT-1) were cultured using medium 199 (Gibco, Grand Island, NY) supplemented with 10% FBS. All *Leishmania* parasites were maintained at 26°C. Approximately 10^6^ parasite cells from culture were extracted for gDNA using GenUP™ gDNA Kit (biotechrabbit, Berlin, Germany) following the manufacturer’s instructions. DNA concentration and quality were determined using a DeNovix DS-11 Spectrophotometer (USA DeNovix Inc., Wilmington, Delaware, USA). In addition, gDNA extracted from *Streptococcus pyogenes*, *Entamoeba histolytica*, *Neisseria gonorrhoeae*, *Escherichia coli*, *Salmonella typhi*, *Corynebacterium diphtheria*, *Plasmodium falciparum*, *Vibrio cholera*, *Trichomonas hominis*, *Shigella flexneri*, *Giardia duodenalis* and human DNA were used as non-*Leishmania* DNA templates. For EDTA anti-coagulated blood samples, the whole blood specimens from HIV-positive patients on antiretroviral therapy (ART) were centrifuged at 900 × *g* for 10 minutes to separate the plasma and buffy coat. A total of 62 buffy coat samples, including 12 *Leishmania*-infected (positive) and 50 non-*Leishmania*-infected samples (negative), were extracted for DNA using the Geneaid^TM^ DNA Isolation Kit (blood) (New Taipei, Taiwan) according to the manufacturer’s protocols.

### Primer design for duplex PCR-NALFIA

The ITS1 region of the SSU-rDNA, including forty-seven *L*. *martiniquensis* and ninety-two *L*. *orientalis* sequences, were retrieved from NCBI to construct consensus sequences of each *Leishmania* species. Forward primers (Universal ITS-F primer; 5´-GCCTTTCCCACATACACAMAC-3’) were designed based on genus-specific sequences, whereas reverse primers (Lm-ITS-288/308R; 5´-AACGAACGGACTTTTCCACTG-3’ and Ls-ITS-233/250R; 5´-CACACCGCCAGAAAAGCC-3’, respectively) were designed based on species-specific sequences that generated 140 and 167 base pair (bp) PCR amplicons of *L*. *martiniquensis* and *L*. *orientalis*, respectively. The specificity of each primer was determined using Primer-BLAST (NCBI). Potential primer dimers and secondary structures were evaluated using OligoCal [35]. The universal ITS-F primer was labeled at the 5’ end with fluorescein isothiocyanate (FITC), including the reverse primers Lm-ITS-288/308R and Ls-ITS-233/250R were labeled at the 5’ end with biotin and digoxigenin, respectively, to identify *Leishmania* species from duplex NALFIA in the lateral-flow device.

### Duplex PCR assays

PCR reaction was performed at a final volume of 25 μL, containing 12.5 μL GoTaq® Green Master Mix 2X (Promega Corporation, USA), 0.2 μM of universal forward primer (duplex reaction), or 0.1 μM of universal forward primer (singleplex reaction) and 0.1 μM of each reverse primer, 1 μL of template DNA, and nuclease-free water to 25 μL. PCR amplification was performed using a thermal cycler (Bio-Rad Laboratories) under the following profile: Initial denaturation at 95°C for 3 min, followed by 35 cycles of denaturation at 95°C for 30 sec, annealing at 60°C for 30 sec, extension at 72°C for 30 sec, and a final extension at 72°C for 5 min. The PCR products were analyzed using electrophoresis in a 2% agarose gel, stained with SYBR® Safe DNA gel stain (Invitrogen, USA), and visualized using a Molecular Imager® ChemiDoc^TM^ XRS+ Imaging System with Imager Lab^TM^5.2.1(Bio-Rad, Hercules, CA, USA).

### Nucleic acid lateral flow immunoassay (NALFIA) device

**Fig 1:**
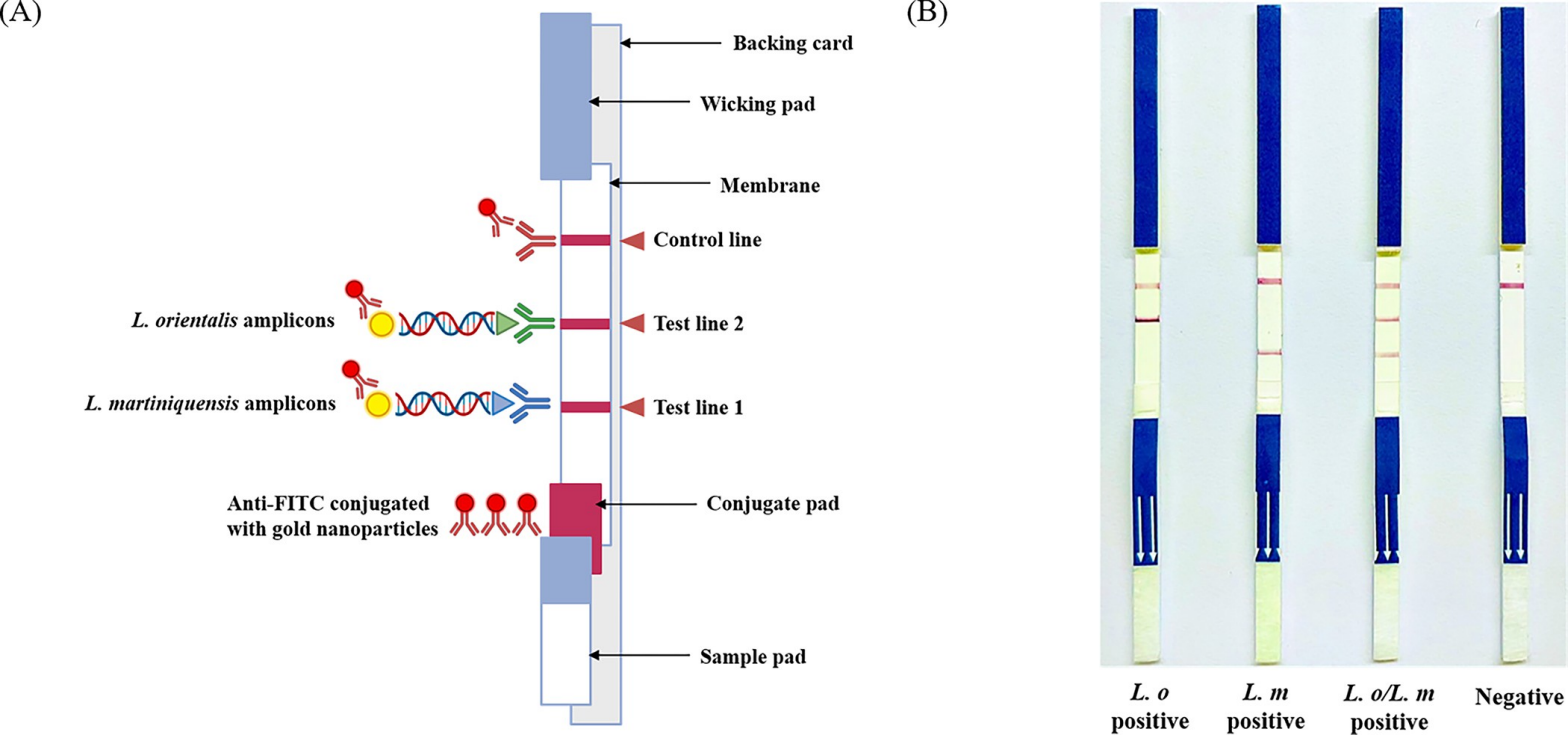
Development and interpretation of the duplex PCR-LFD for detecting *Leishmania martiniquensis* and *Leishmania orientalis*. (A) Schematic representation of the duplex PCR-LFD. The test strip consisted of specific test lines for capturing amplicons of *L*. *martiniquensis* (Test line 1) and *L*. *orientalis* (Test line 2), with a control line to ensure assay validity. (B) The test strips are representative of positive and negative results. The specific bands corresponding to the presence of *L*. *orientalis* (*L*. *o* positive), *L*. *martiniquensis* (*L*. *m* positive), and both *L*. *orientalis* and *L*. *martiniquensis* (*L*. *o*/*L*. *m* positive) amplicons are shown. For the absence of any target amplicons, only the control line is observed (negative).

The duplex NALFIA in LFD format was constructed by assembling dipsticks with, in addition to a control line, two distinct test lines specific to *L*. *martiniquensis* and *L*. *orientalis*, as shown in Fig 1. Kestrel Bioscience Thailand Co. Ltd performed the assembly. In brief, the LFD consisted of four components: sample pad, conjugate pad, absorbent pad, and nitrocellulose membrane. Within the conjugate pad, anti-FITC conjugated gold nanoparticles (AuNPs) were incorporated to interact with the FITC-labeled end of the double-labeled amplicons. The nitrocellulose membrane hosted immobilized capture reagents at the reaction site, encompassing anti-biotin positioned as test line 1 for *L*. *martiniquensis* amplicons and anti-digoxigenin set as test line 2 for *L*. *orientalis* amplicons. The conjugate control line (C) featured an immobilized probe complementary to excess anti-FITC conjugated AuNPs, serving as an indicator of the functionality of the LFD.

### Specificity and limit of detection (LoD) of duplex PCR assay

The cross-reactivity of the designed primer was first evaluated by singleplex reaction using individual DNA templates from *L*. *martiniquensis* and *L*. *orientalis* before being further evaluated by duplex reaction using a mixture of DNA from both *Leishmania* species. The specificity of the duplex PCR was further assessed using *L*. *donovani*, *L*. *infantum*, *S*. *pyogenes*, *E*. *histolytica*, *N*. *gonorrhoeae*, *E*. *coli*, *S*. *typhi*, *C*. *diphtheria*, *P*. *falciparum*, *V*. *cholera*, *T*. *hominis*, *S*. *flexneri*, *G*. *duodenalis*, and human DNA.

To confirm the specificity of the duplex PCR reaction, standard melt curve analysis was performed by qPCR to determine species-specific melting point values of PCR amplicons between *L*. *martiniquensis* and *L*. *orientalis*. Each reaction mixture was prepared in a total volume of 20 μL and consisted of 4X CAPITAL™ qPCR Green Master Mix (Biotechrabbit GmbH, Germany) and 2 μL of DNA template using a Rotor Gene-Q (Qiagen, Germany). The qPCR profile was an initial denaturation at 95°C for 3 min, followed by 40 cycles of 95°C for 20 sec and 60°C for 30 sec, followed by temperature increment from 55°C to 95°C at a rate of 1°C per sec.

The gDNA extracted from 10^6^ cells of *L*. *martiniquensis* or *L*. *orientalis* was serially diluted to 1,000, 500, 100, 50, 10, 5, 1, 0.5, and 0.1 parasites/μL to determine the limit of detection of the duplex PCR assay. Subsequently, one μL of the serially diluted gDNA of each *Leishmania* species and a mixture of *L*. *martiniquensis* and *L*. *orientalis* was used as a DNA template. The experiments were performed in triplicate with nuclease-free water as a negative control.

### Specificity and limit of detection (LoD) of PCR-LFD using NALFIA device

To assess the specificity of PCR-LFD using NALFIA device, the cross-reactivity of the duplex PCR was prepared as described previously using *L*. *donovani*, *L*. *infantum*, and *S*. *pyogenes*, *E*. *histolytica*, *N*. *gonorrhoeae*, *E*. *coli*, *S*. *typhi*, *C*. *diphtheria*, *P*. *falciparum*, *V*. *cholera*, *T*. *hominis*, *S*. *flexneri*, *G*. *duodenalis* and human DNA. In addition to that duplex PCR assay, each *Leishmania* species (*L*. *martiniquensis* and *L*. *orientalis*) and a mixture of *L*. *martiniquensis* and *L*. *orientalis* were used as positive controls. Following PCR amplification, 5 μL of the duplex PCR reaction was pipetted and mixed with 70 μL of LFD running buffer. The reaction mixture was then applied to the sample pad of the NALFIA device and evaluated for the result by the naked eye within 15 minutes. A positive result is interpreted by clear color on both control and test lines, with test line 1 indicating *L*. *martiniquensis* amplicons and test line 2 marking *L*. *orientalis* amplicons. Conversely, a negative result was indicated by color solely on the control line. The flow assay was considered invalid if no color was visible on the control line.

The limit of detection (LoD) of PCR-LFD was determined using ten-fold serial dilutions of each *Leishmania* species (*L*. *martiniquensis* and *L*. *orientalis*) and the mixture of *L*. *martiniquensis* and *L*. *orientalis* ranging from 10^5^ to 10^−5^ parasites/μL that were initially amplified using duplex PCR to evaluate the suitable condition of duplex PCR for single-target and mixed-target gDNA.

### Evaluation of sensitivity and specificity of PCR-LFD

The sensitivity and specificity of PCR-LFD were evaluated using 62 genomic DNA samples from the buffy coat, consisting of 12 confirmed asymptomatic visceral leishmaniasis, including *L*. *martiniquensis* (*n* = 6) and *L*. *orientalis* (*n* = 6) and 50 uninfected cases. Diagnosis of VL was confirmed when nested PCR targeting the ITS1 region of the rRNA gene was positive, and the DNA sequence of the PCR amplicons was identical to *Leishmania*’s DNA. Nested PCR results were considered a reference standard method due to the unavailability of any gold standard used to validate and evaluate the sensitivity and specificity of PCR-LFD [13, 29, 36]. The sensitivity, specificity, positive predictive value (PPV), and negative predictive value (NPV) of PCR-LFD were calculated along with their 95% confidence intervals. Furthermore, the strength of agreement between the nested PCR and PCR-LFD detection methods was assessed using the kappa statistical test, with a significance threshold of *P* < 0.05 and a 95% confidence interval.

## Results

### Specificity and limit of detection of duplex PCR amplification

**Fig 2:**
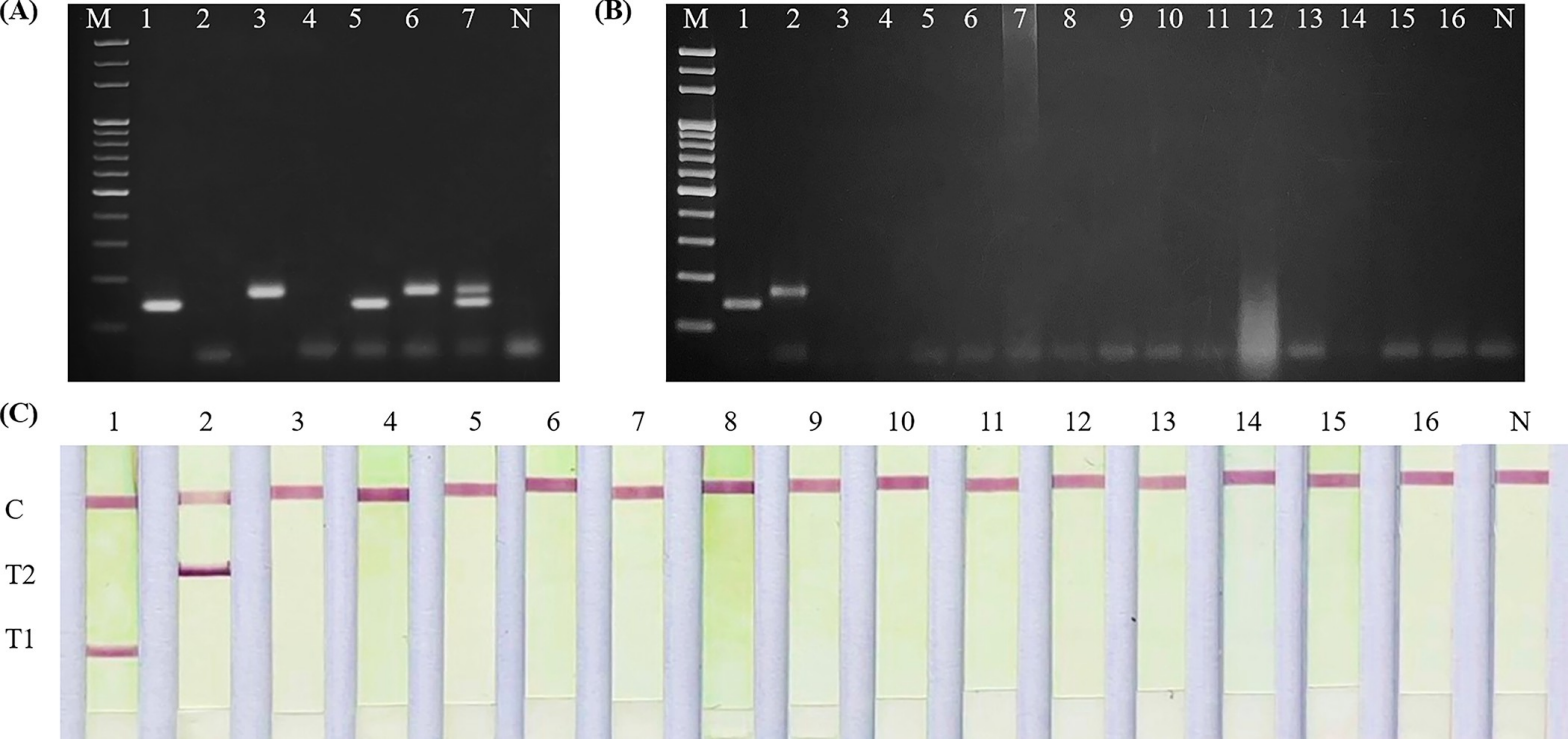
Specificity of the duplex PCR and PCR-LFD for identification of *L*. *martiniquensis* and *L*. *orientalis*. (A) Singleplex PCR and duplex PCR reactions. M: 100 bp DNA ladder; Lane 1–2: Singleplex PCR using universal ITS-F and Lm-ITS-288/308R tested with *L*. *martiniquensis* and *L*. *orientalis* DNA templates, respectively. Lane 3–4: Singleplex PCR using universal ITS-F and Ls-ITS-233/250R tested with DNA templates from *L*. *orientalis* and *L*. *martiniquensis*, respectively. Lane 5–7: Duplex PCR reactions tested with DNA templates from *L*. *martiniquensis*, *L*. *orientalis* and mixed DNA templates from both species. N: Non-template control. (B) and (C) Cross-reactivity of duplex PCR and PCR-LFD, respectively; Lane 1–16: *L*. *martiniquensis* (1), *L*. *orientalis* (2), *L*. *donovani* (3), *L*. *infantum* (4), *Streptococcus pyogenes* (5), *Entamoeba* sp. (6), *Neisseria gonorrhoeae* (7), *Escherichia coli* (8), *Salmonella typhi* (9), *Corynebacterium diphtheria* (10), *Plasmodium falciparum* (11), *Vibrio cholera* (12), *Trichomonas hominis* (13), *Shigella flexneri* (14), *Giardia duodenalis* (15), Human (16). N: Non-template control.

In singleplex reactions, the universal ITS-F and Lm-ITS-288/308R primers specifically amplified fragments from *L*. *martiniquensis* gDNA, displaying no cross-reactivity with *L*. *orientalis*. Similarly, the universal ITS-F and Ls-ITS-233/250R primers generated DNA fragments specific for *L*. *orientalis*, with no cross-reactivity to *L*. *martiniquensis*. After combining all three primers in the duplex reaction, there was no change in the specificity to amplify target sites of *L*. *martiniquensis* and *L*. *orientalis* using either single gDNA or mixed gDNA of both *Leishmania* species. Visualization of gel electrophoresis confirmed the correct amplicon sizes of distinct bands, which were 140 bp for *L*. *martiniquensis* and 167 bp for *L*. *orientalis* (Fig 2A). Notably, no cross-amplified product was detected from non-*Leishmania* DNA, thus confirming the exceptional specificity of the assay (Fig 2B).

Under the duplex qPCR conditions, PCR amplicons of *L*. *martiniquensis* exhibited a length of 140 bp with a melting temperature of 86.7°C. In comparison, the PCR amplicons of *L*. *orientalis* were 167 bp with a melting point of 84.5°C. The precise difference in amplitude of melting temperature suggested the specific amplification of the duplex PCR products between *L*. *martiniquensis* and *L*. *orientalis* (S1 Fig).

**Fig 3:**
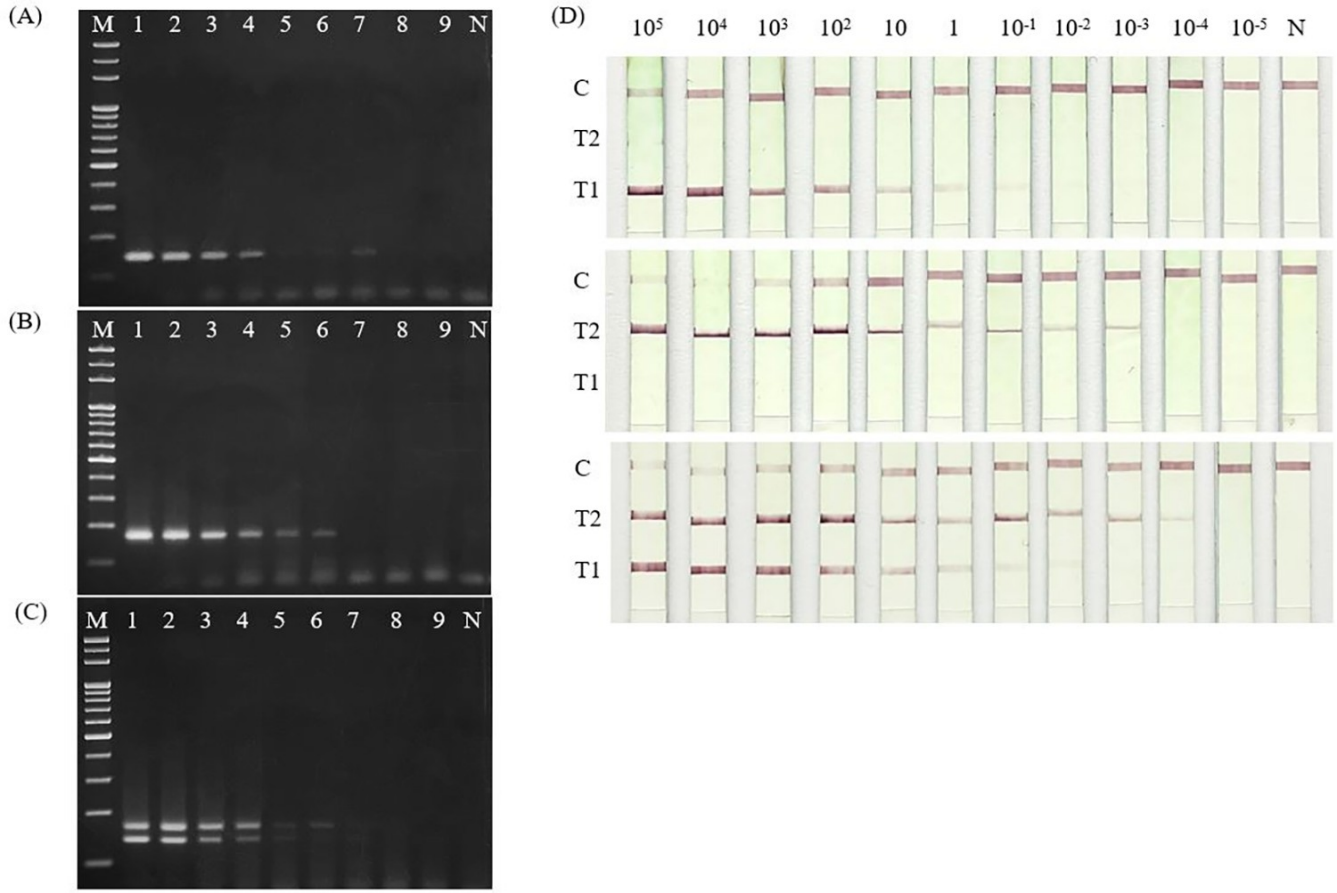
Limits of duplex PCR and PCR-LFD detection based on genomic DNA of *L*. *martiniquensis* and *L*. *orientalis*. (A) Limit of detection of duplex PCR using *L*. *martiniquensis* DNA. (B) Limit of detection of duplex PCR using *L*. *orientalis* DNA. (C) Limit of detection of duplex PCR using a mixture of *L*. *martiniquensis* and *L*. *orientalis* DNA. Lanes 1–9 of (A-C): the amount of DNA template was 1000, 500, 100, 50, 10, 5, 1, 0.5, and 0.1 parasite/μL, respectively. N: Non-template control. (D) Limit of detection of PCR-LFD using tenfold serially diluted DNA templates (10^5^−10^−5^ parasite/μL) including *L*. *martiniquensis* DNA (top panel), *L*. *orientalis* DNA (middle panel), and 1:1 ratio mixture of *L*. *martiniquensis* and *L*. *orientalis* DNA (bottom panel).

The LoD was as low as one parasite/μL for *L*. *martiniquensis* and five parasite/μL *L*. *orientalis* (Fig 3A and 3B). Additionally, the LoD value of this assay remained consistent when using either single gDNA or mixed gDNA of both *Leishmania* species (Fig 3A–3C). Thus, our designed primers could be simultaneously performed in singleplex and duplex PCR to detect either *L*. *martiniquensis* or *L*. *orientalis* in a reaction mixture with highly similar sensitivity.

### Specificity and limit of detection of PCR-LFD assay

The PCR-LFD was shown to specifically detect and accurately differentiate either *L*. *martiniquensis* or *L*. *orientalis*. The device strip displayed a pink color of AuNPs on the test band, indicating the presence of the targeted species. No cross-amplification and cross-reactivity were observed on the device strips when non-*Leishmania* PCR amplicons were used and tested, suggesting that the specificity of the PCR-LFD was high (Fig 2C).

The LoD of the PCR-LFD was determined using 10-fold serial dilution gDNA of both *Leishmania* species. Different detection sensitivity was observed in PCR reaction mixtures of each single gDNA serial dilution preparation. It showed that the LoD of *L*. *martiniquensis* was 10^−1^ parasite/μL, whereas LoD of *L*. *orientalis* was 10^−3^ parasite/μL. In addition, the device strip could simultaneously detect the PCR products from mixed gDNA of *L*. *martiniquensis* and *L*. *orientalis* at 10^−2^ parasite/μL and 10^−4^ parasite/μL, respectively (Fig 3D).

### Evaluation of sensitivity and specificity of PCR-LFD using clinical specimens

Sixty-two extracted DNA from buffy coats from patients with HIV were used to evaluate the sensitivity and specificity of PCR-LFD. Among these samples, the presentation of *Leishmania* DNA was previously confirmed by the conventional nested PCR method. Six samples were positive for *L*. *martiniquensis*, whereas six were positive for *L*. *orientalis*. The remaining 50 samples were negative for any visceral *Leishmania* species. None of *Leishmania*’s positive samples revealed mixed infection. One microliter of gDNA was used to amplify duplex PCR before colorimetric visualized examination using our lateral flow device strip based on nucleic acid lateral flow immunoassay for duplex detection of *L*. *martiniquensis* and *L*. *orientalis*. A comparative analysis of the results against the conventional nested PCR is shown in Table 1. The results revealed that the PCR-LFD assay highlighted a sensitivity of 100% (*n* = 6; 95% CI, 54.1% to 100.0%) for *L*. *martiniquensis* detection and 83.33% sensitivity (*n* = 6; 95% CI, 35.9% to 99.6%) for *L*. *orientalis* detection. The PCR-LFD demonstrated the same specificity as high as 100% (*n* = 50; 95% CI, 92.9% to 100.00%) for detecting *L*. *martiniquensis* and *L*. *orientalis*. The level of agreement between the nested PCR and PCR-LFD was determined using the Κappa statistics, yielding a value of 0.948, indicating almost perfect agreement between these two methods.

**Table 1:** Sensitivity and specificity of PCR-LFD assay for duplex detection of *L*. *martiniquensis* and *L*. *orientalis* in asymptomatic patients with HIV.

PCR-LFD assay	No. (*n*) of samples with nested PCR^*a*^		(%) Sensitivity (95% CI)	(%) Specificity (95% CI)	(%) PPV (95% CI)	(%) NPV (95% CI)
	Positive	Negative				
*L*. *martiniquensis*						
Positive	6	0	100% (54.1%- 100%)	100.0% (92.9%-100.0%)	100.0% (0.0%-0.0%)	100.0% (0.0%-0.0%)
Negative	0	50				
*L*. *orientalis*						
Positive	5	0	83.3% (35.9%-99.6%)	100.0% (92.9%-100.0%)	100.0% (0.0%-0.0%)	98.0% (89.3%-99.7%)
Negative	1	50				

^*a*^The total number of positive results was based on nested PCR (reference standard method).

## Discussion

Leishmaniasis, caused by *L*. *martiniquensis* and *L*. *orientalis*, constitutes a significant public health concern due to its increased emergence in diverse regions, particularly among high-risk groups like patients with HIV/AIDS. Precise and accurate species identification is critical for effective disease diagnosis, epidemiological understanding, and the development of control strategies. In this study, we have introduced a duplex PCR assay, targeted on the SSU-rRNA region, complemented with LFD, enabling the specific identification of *L*. *martiniquensis* and *L*. *orientalis*, endemic species in Thailand.

SSU-rRNA sequences are typically preferred for species identification across various organisms, including *Leishmania* species. Due to its genetic information, the SSU-rRNA phylogenetic tree analysis revealed that *L*. *martiniquensis* (*L*. *siamensis* lineages PG) and *L*. *orientalis* (*L*. *siamensis* lineage TR) were closely related and grouped apart from other *Leishmania* species [37]. The high conservation of transcribed SSU-rRNA regions is more suitable for distinguishing upper taxonomic levels, such as between genera or subgenera. Still, it could lack the information specific to differentiate closely related species compared with the ITS1 region. This study found that ITS1 sequences exhibit sufficient intraspecific variation, making them suitable for accurately identifying *L*. *martiniquensis* and *L*. *orientalis*. It highlights the suitability of ITS1 as a genetic marker for accurate species identification within the *Leishmania* genus.

This present study developed a duplex PCR assay to identify and differentiate between *L*. *martiniquensis* and *L*. *orientalis*. Three newly designed primers targeting the ITS1 region generated distinctive PCR products of 140 bp and 167 bp for *L*. *martiniquensis* and *L*. *orientalis*, respectively, allowing their distinguishability using gel electrophoresis. The relatively short amplicons also make them suitable for real-time PCR techniques, with the recommended size typically ranging from 50 to 150 base pairs.

For *L*. *martiniquensis*, the duplex PCR technique could detect as low as one parasite/μL. It demonstrates a superior sensitivity compared to the previous qPCR-ITS1 assay, where the reported detection sensitivity was 50 parasites/μL [38]. Similarly, the duplex PCR-ITS1 exhibited remarkable sensitivity, as evidenced by its ability to detect as low as 5 parasite/μL of *L*. *orientalis*. The sensitivity level was comparable to the previous PCR-ITS1 assay, which was reported as the most sensitive method for detecting *L*. *orientalis* [26]. The high sensitivity of the newly developed duplex PCR-ITS1 assay is attributed to the high number of rRNA gene copies, ranging from 20 to 200 copies, present in the *Leishmania* genome [39]. These findings highlight the potential of duplex PCR-ITS1 assay as an effective and practical tool for species discrimination in leishmaniasis cases.

Considerations of cross-reactivity and specificity are paramount in duplex PCR due to the intricate interactions among multiple primer sets and target sequences. In this context, the newly designed primers were thoroughly examined for specificity by assessing their potential for cross-amplification with other major viscerotropic *Leishmania* species, including *L*. *donovani* and *L*. *infantum*, as well as other known organisms. The results confirmed the assay’s high specificity, ensuring reliable species identification and discrimination.

Simultaneous detection using duplex PCR proves to be a time and cost-efficient approach, making it suitable for large-scale screening and surveillance. This study has further integrated LFD strip tests with PCR to enhance its diagnostic capabilities, providing an even more effective approach. The LFD utilizes an immunochromatographic assay to detect amplified PCR products within minutes visually, offering rapid results and requiring minimal equipment and expertise. The analytical sensitivity of the present PCR-LFD was compared with the results from agarose gel electrophoresis using SYBR® Safe DNA gel stain for both single and mixed DNA templates of *L*. *martiniquensis* and *L*. *orientalis*. The results indicated that the PCR-LFD demonstrated a remarkable simultaneous detection limit of 10^−2^ parasite/μL for *L*. *martiniquensis* and 10^−4^ parasite/μL for *L*. *orientalis*, which is at least 100–10,000-fold higher than the analytical sensitivity of agarose gel electrophoresis, consistent with previous research findings [33, 40] The assay’s sensitivity underscores its potential utility in identifying asymptomatic cases and monitoring disease trends. Apart from its superior sensitivity, the LFD, as a PCR readout system, offers several advantages over gel electrophoresis. It provides rapid results within minutes and requires minimal equipment and expertise. This robust diagnostic tool is also practical for large-scale screening and surveillance capabilities, essential for tracking and managing diseases like leishmaniasis.

The duplex PCR-LFD device, a novel tool in the field of leishmaniasis research, was validated using DNA samples from non-*Leishmania*-infected individuals and confirmed *Leishmania*-infected patients. With the limited availability of infected samples taken to analyze, the diversity and variability within the population may not be fully represented, leading to less precision estimation of assay sensitivity. Indeed, it is essential to note that the sensitivity of PCR-LFD is commonly reported to be slightly lower than that of nested PCR or qPCR for detecting target DNA sequences [41, 42], which could explain the false-negative result. The PCR-LFD demonstrated high specificity for detecting both *L*. *martiniquensis* and *L*. *orientalis*, with a specificity of 100% for each species. This high specificity was evident by obtaining negative results from all 50 non-*Leishmania*-infected samples, indicating that the assay accurately distinguished between *Leishmania*-infected and non-infected samples among asymptomatic patients with HIV. Due to the unavailability of co-infected cases, it is infeasible to identify any potential limitations or biases of multiple detection of specific species for simultaneous detection of *Leishmania* species using the duplex PCR-LFD in a clinical sample. Further validation with a more extensive clinical sample size and diverse *Leishmania* species would enhance this diagnostic tool’s broader application and utility.

The need for a capable tool for species discrimination becomes increasingly valuable when addressing emerging organisms like *L*. *martiniquensis* and *L*. *orientalis*, in which basic information is still lacking. These different species, even though they exhibit similar clinical manifestations, may possess distinct biological traits, such as growth rate, population density, drug susceptibility profiles, and interactions with vectors and reservoir hosts. As visible in other viscerotropic species, *L*. *donovani* is considered to be more aggressive than *L*. *infantum* due to the poorer treatment responses [43]. In addition, the transmission of *L*. *donovani* typically relies on anthroponotic transmission, while *L*. *infantum* is frequently associated with zoonotic transmission [44].

Moreover, considering their geographical distribution, *L*. *donovani* predominantly spreads in East Africa and the Indian subcontinent, whereas *L*. *infantum* exhibits a higher prevalence in the Mediterranean region and Latin America [45]. The PCR-LFD diagnostic tool developed in this study enhances the accuracy and efficiency of diagnosing leishmaniasis caused by *L*. *martiniquensis* and *L*. *orientalis*, predominantly detected in HIV patients. It plays a pivotal role in advancing epidemiological research and control efforts, especially in regions where HIV/AIDS prevalence is high. It offers valuable insights into the distribution of these two *Leishmania* species, disease trend monitoring, and rapid outbreak response. By supporting various research studies and surveillance programs, this tool could contribute to a better understanding of leishmaniasis epidemiology and holds great promise for effective management and prevention of leishmaniasis in Thailand and other affected regions.

## Conclusion

Our findings highlight the potential of the duplex PCR-LFD assay as a combination of sensitivity, specificity, rapidity, and user-friendliness, making it a valuable tool for detecting and identifying *L*. *martiniquensis* and *L*. *orientalis* infections, particularly in patients with HIV. This approach could hold a high prospect for ongoing leishmaniasis research and control efforts, providing a practical tool for large-scale screening and surveillance. Ultimately, it will contribute to more effective disease management and help address the pressing public health concern of leishmaniasis.

## Supporting information

S1 Fig Melting curve assay for the detection of *L*. *martiniquensis* and *L*. *orientalis*. (A) Amplification curves. (B) Melting curves. Melting temperatures of the DNA amplicons amplified by species-specific duplex primers for detecting *L*. *martiniquensis* and *L*. *orientalis* were indicated in parenthesis. NTC: non-template control.
(TIFF)

S1 Raw image The original gel images underlying PCR analyses. (PDF)
