# Supplementary figures and images for: Development of nucleic acid lateral flow immunoassay for duplex detection of Leishmania martiniquensis and Leishmania orientalis in asymptomatic patients with HIV
Source: PLoS One. 2024 Aug 26;19(8):e0307601. doi: 10.1371/journal.pone.0307601 (PMC11346928; doi:10.1371/journal.pone.0307601)

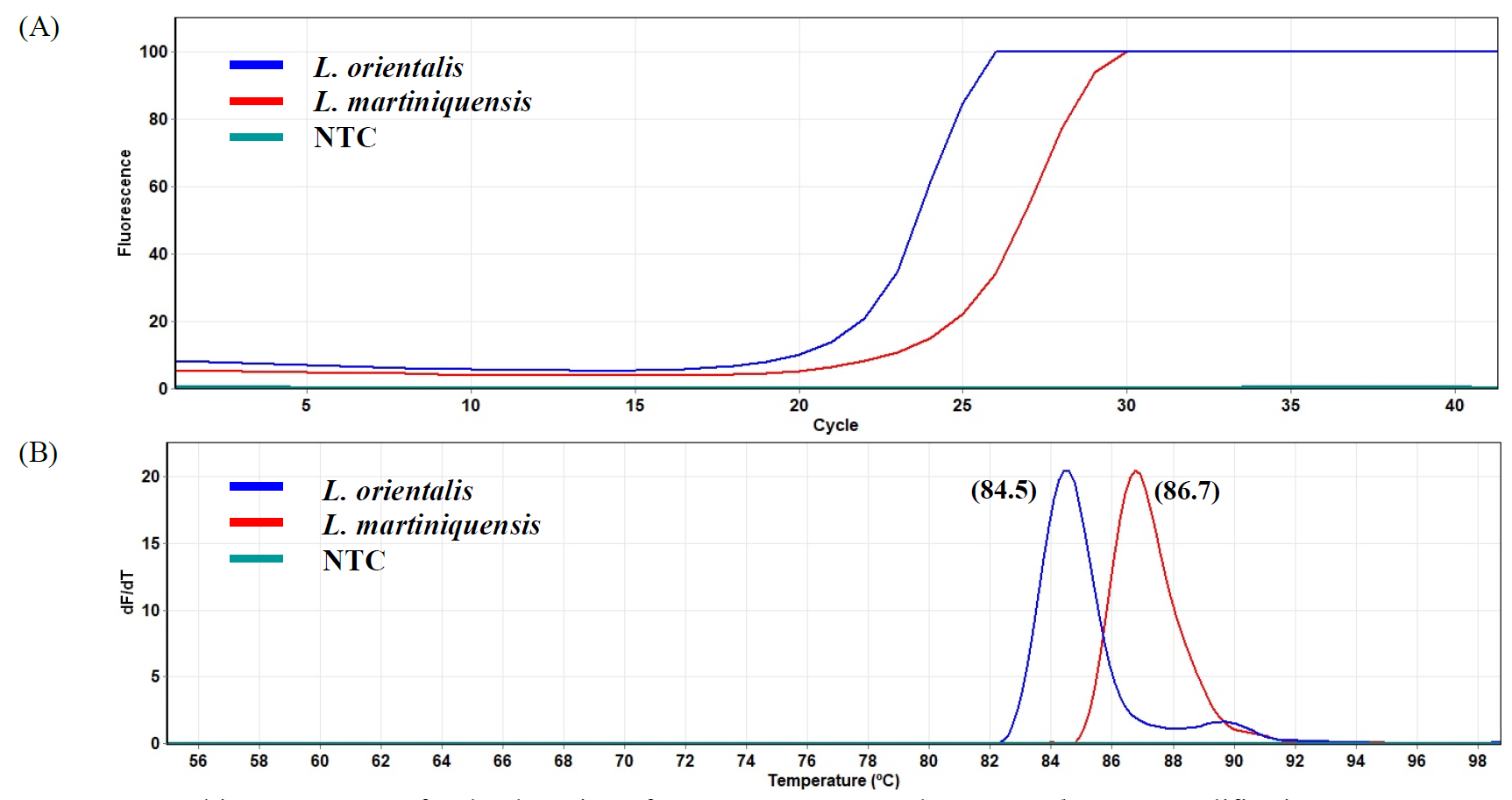

Supplement: S1 Fig — (A) Amplification curves. (B) Melting curves. Melting temperatures of the DNA amplicons amplified by species-specific duplex primers for detecting L. martiniquensis and L. orientalis were indicated in parenthesis. NTC: non-template control. (TIFF) [file pone.0307601.s001.tiff]
