# Supplementary material for: Development of nucleic acid lateral flow immunoassay for duplex detection of Leishmania martiniquensis and Leishmania orientalis in asymptomatic patients with HIV
Source: PLoS One. 2024 Aug 26;19(8):e0307601. doi: 10.1371/journal.pone.0307601 (PMC11346928; doi:10.1371/journal.pone.0307601)
Supplement: S1 Raw image — (PDF) [file pone.0307601.s002.pdf]

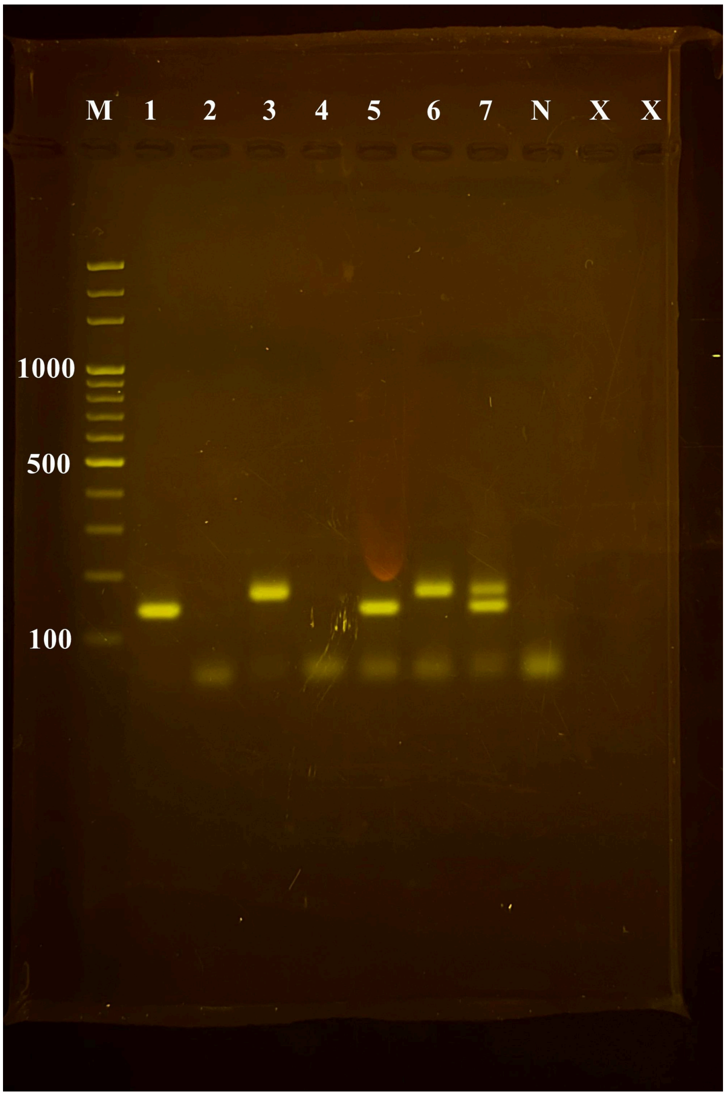

Fig 2A. Singleplex and duplex PCR reaction specificity

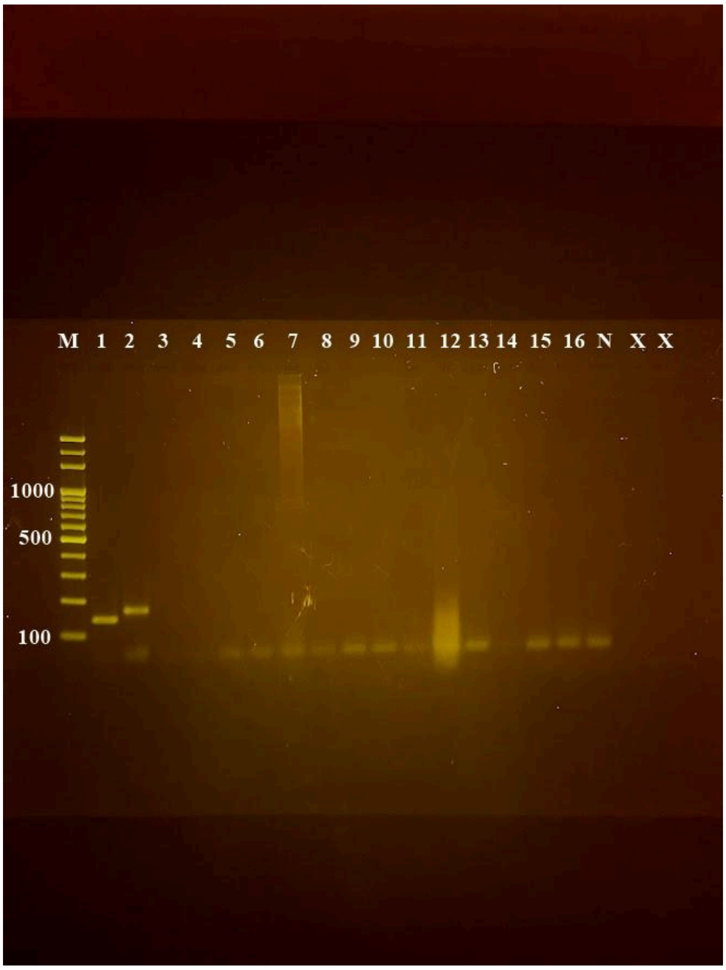

Fig 2B. Cross-reactivity of duplex PCR reaction

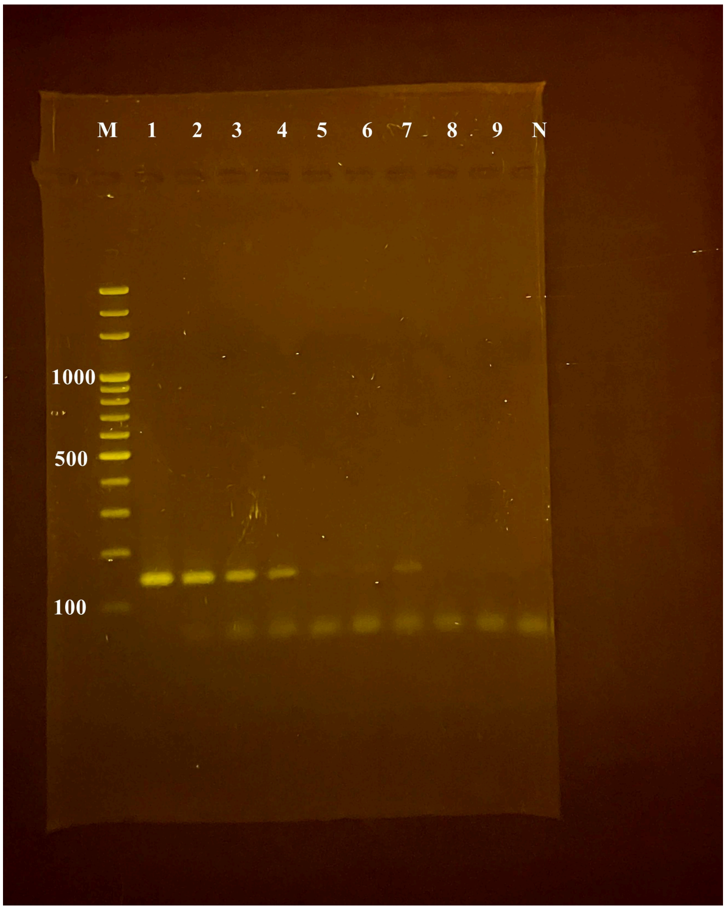

Fig 3A. Limit of duplex PCR detection using *L. martiniquensis* DNA

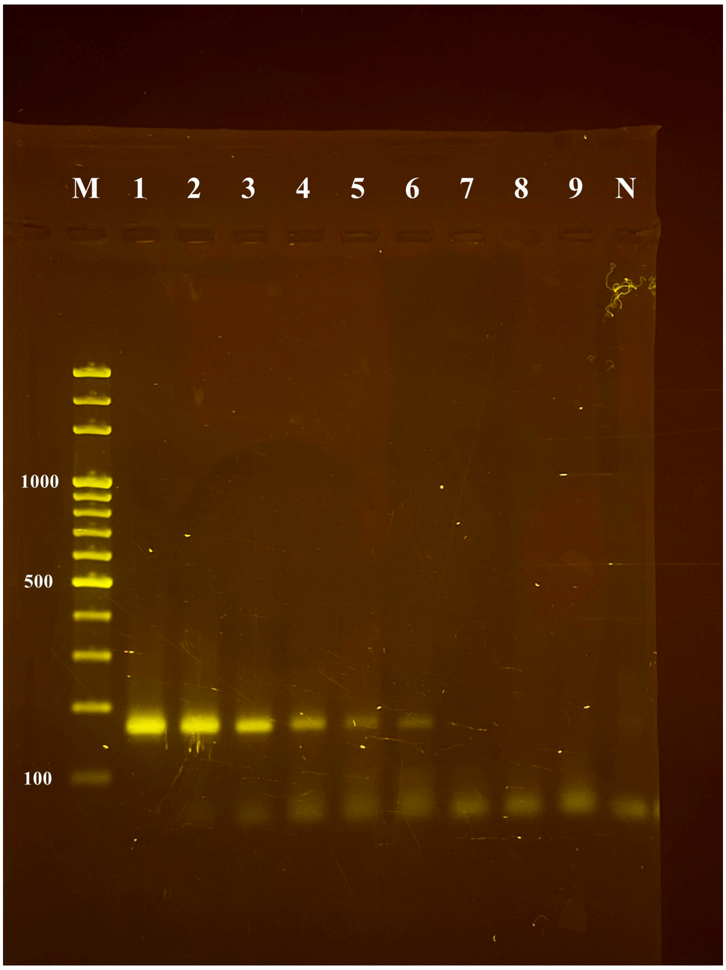

Fig 3B. Limit of duplex PCR detection using *L. orientalis* DNA

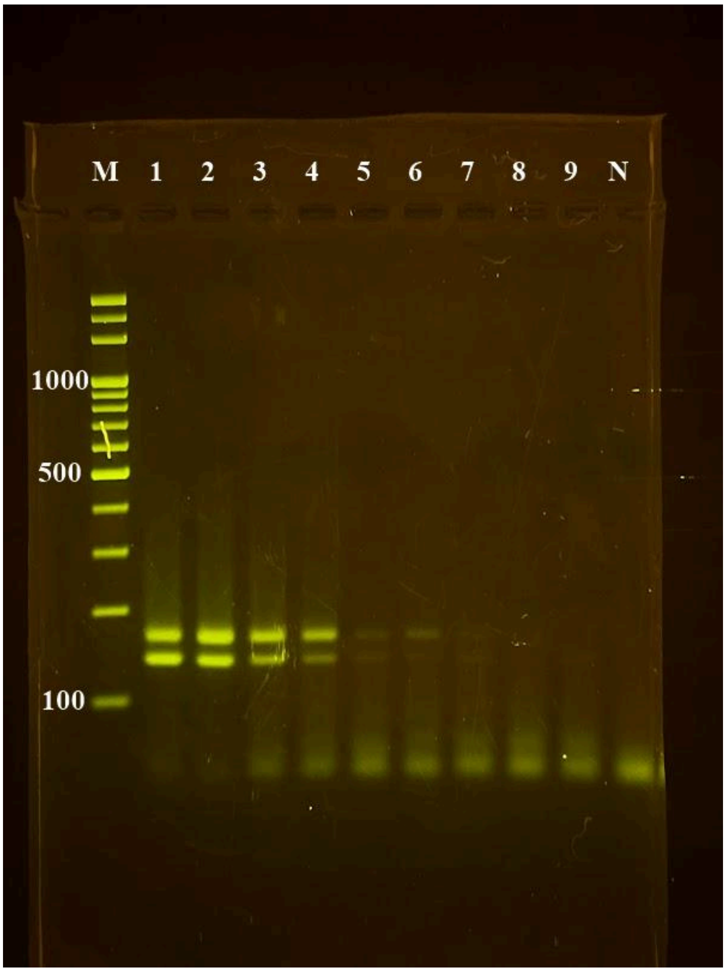

Fig 3C. Limit of duplex PCR detection utilizing a mixture of *L. martiniquensis* and *L. oreintalis* DNA
